# Symmetry-Adapted Rotator Functions for Molecules in Cylindrical Confinement

**DOI:** 10.3390/ijms12010317

**Published:** 2011-01-13

**Authors:** Bart Verberck

**Affiliations:** Departement Fysica, Universiteit Antwerpen, Groenenborgerlaan 171, B-2020 Antwerpen, Belgium; E-Mail: bart.verberck@ua.ac.be

**Keywords:** molecular symmetry, symmetry-adapted rotator functions, cylindrical confinement

## Abstract

We present a general description of the formalism of symmetry-adapted rotator functions (SARFs) for molecules in cylindrical confinement. Molecules are considered as clusters of interaction centers (ICs), can have any symmetry, and can display different types of ICs. Cylindrical confinement can be realized by encapsulation in a carbon nanotube (CNT). The potential energy of a molecule surrounded by a CNT can be calculated by evaluating a limited number of terms of an expansion into SARFs, which offers a significant reduction of the computation time. Optimal molecular orientations can be deduced from the resulting potential energy landscape. Examples, including the case of a molecule with cubic symmetry inside a CNT, are discussed.

## 1. Introduction

Symmetry plays an extremely important role in nature. Accordingly, the mathematics of symmetry is embedded in many aspects of theoretical physics. In particular, many concepts from group theory have been applied to describe the crystal structure of solids.

Molecular crystals combine the symmetry of a crystal lattice with molecular symmetries (for a review, see Ref. [1]). Various physical properties of molecular crystals can be described in terms of functions adapted to the molecules’ symmetry and the symmetry of the sites they occupy in the crystal lattice: symmetry-adapted rotator functions (SARFs). They were first introduced by James and Keenan for the description of solid heavy methane, CD_4_ [2]. General descriptions have been elaborated by Yvinec and Pick [3] and Michel and Parlinski [4].

Traditionally, SARFs have been used to describe three-dimensional lattices. However, in recent years, molecules have been successfully inserted into carbon nanotubes (CNTs), the internal hollow space of which provides cylindrical confinement. The first reported synthesis of such a system (called “nanopeapod”) featured C_60_ molecules encapsulated in a CNT [5]. The SARFs for a C_60_ molecule in cylindrical confinement were developed afterwards [6,7]. By now, SARFs for cylindrical site symmetry have been developed for C_60_, C_70_ and C_80_ peapods, each featuring different molecular symmetries (*I_h_* [8], *D*_5_*_h_* [9] and *D*_5_*_d_* [10], respectively).

The purpose of the present paper is to provide a general description of the construction of SARFs for molecules of any symmetry in cylindrical confinement. First, we present a pedestrian approach to the example of a C_60_ peapod: we show how the potential energy of the C_60_ molecule, positioned on the long axis of a CNT, can be expanded into a series of SARFs. We then discuss the resulting formulas, and extend the potential model used for calculating the interaction energy of a C_60_ molecule and the surrounding CNT. This is followed by the general construction of SARFs. While the main goal is to focus on the mathematical formalism behind SARFs, we will also show potential energy landscapes for various tube radii (“nanotube fields”) and point to the associated optimal molecular orientations. The practical advantage of SARFs expansions is discussed. In addition, we provide an original example with cubic molecular symmetry.

## 2. Theoretical Formalism

### 2.1. Example #1

**Figure 1:**
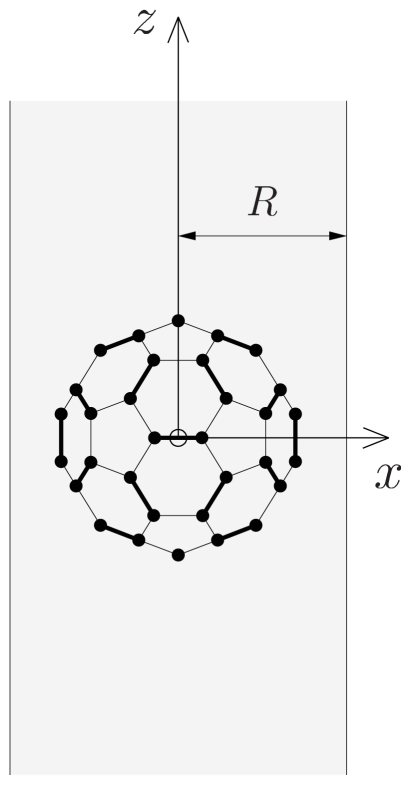
A C_60_ molecule in a CNT with radius *R*; the molecule is positioned on the tube’s long axis (*z*-axis). Double bonds are shown thicker than single bonds; the depicted molecular orientation is the so-called standard orientation.

It is instructive to introduce the formalism of SARFs for cylindrical confinement by elaborating a concrete example. We consider a C_60_ molecule encapsulated in a CNT with its center of mass on the tube’s long axis (Figure 1). We treat the molecule as a rigid cluster of 60 carbon atoms, labeled Λ_a_ = 1*, . . . ,* 60, and the nanotube as a homogeneous cylindrical density distribution *n*(*r⃗*) of carbon atoms (the subscript _a_ stands for atom). The interaction energy then reads

(1) $$V=\sum_{\Lambda_{\text{a}}=1}^{60}\int d\vec{r}n(\vec{r})v(|\vec{r}-{\vec{r}}_{\Lambda_{\text{a}}}|)$$

where *r⃗*_Λ_a__ = (*x*_Λ_a__*, y*_Λ_a__*, z*_Λ_a__) is the position vector of atom Λ_a_ of the C_60_ molecule. The function *v*(*d*) is the pair potential function giving the energy of two interacting centers (a carbon atom of the molecule and a carbon atom of the tube) at a distance *d* apart. Its precise form is not essential at this moment. For a tube of radius *R*, *n*(*r⃗*)*dr⃗* = *σδ*(*ρ* − *R*)*ρdρd*Φ*dZ*, and *V* becomes

(2) $$\begin{array}{l} V=\sigma \sum_{\Lambda_{\text{a}}=1}^{60}\int_{0}^{\infty }\rho d\rho \int_{0}^{2\pi }d\varphi \int_{-\infty }^{+\infty }dZ\;\delta (\rho -R)v(|\vec{r}-{\vec{r}}_{\Lambda_{\text{a}}}|)\\ =\sigma R\sum_{\Lambda_{\text{a}}=1}^{60}\int_{0}^{2\pi }d\Phi \int_{-\infty }^{+\infty }dZ\;v(|{\vec{r}}_{R}-{\vec{r}}_{\Lambda_{\text{a}}}|) \end{array}$$

Here, cylindrical coordinates (*ρ,*Φ*,Z*) have been introduced so that *r⃗* = (*ρ* cos Φ*, ρ* sin Φ*, Z*), and *r⃗_R_* = (*R*cos Φ*, R* sin Φ*, Z*). The quantity *σ* is the tube’s surface density, the value for rolled-up graphene sheets (CNTs) is 0.372 Å^−2^.

We have not yet specified the molecule’s orientation. Let us introduce a reference orientation for the C_60_ molecule, we choose it to be the orientation where two-fold symmetry axes coincide with the coordinate axes—the so-called standard orientation (Figure 1). The molecule’s center of mass coincides with the origin and the *z*-axis is chosen to coincide with the tube’s long axis. Molecular rotations can now be specified with respect to this standard orientation. Using the Euler angle convention of Ref. [11], any rotation can be described as the succession of three Euler rotations: (i) a rotation ℜ*_z_*(*α*) over 0 ≤ *α <* 2*π* about the *z*-axis, followed by (ii) a rotation ℜ*_y_*(*β*) over 0 ≤ *β* ≤ *π* about the *y*-axis, and finally (iii) a rotation ℜ*_z_*(*γ*) over 0 ≤ *γ <* 2*π* about the *z*-axis again. The *x*-, *y*- and *z*-axes are kept fixed. Note that *α* = *β* = *γ* = 0 then corresponds to the standard (reference) orientation. With the convention of Ref. [11], any coordinate function *f*(*r⃗*) is transformed as ℜ (*α, β, γ*)*f*(*r⃗* ) = *f*(ℜ^−1^(*α, β, γ*) *r⃗* ), where ℜ (*α, β, γ*) = ℜ*_z_*(*γ*) ℜ*_y_*(*β*) ℜ*_z_*(*α*). Applying this to the pair potentials *v*, essentially functions of the atomic positions *r⃗*_Λ_a__, results in the following explicit expression for the molecule-tube interaction energy *V* (*α, β, γ*) for a rotated molecule:

(3) $$V(\alpha ,\beta ,\gamma )\equiv R(\alpha ,\beta ,\gamma )V=\sigma R\sum_{\Lambda_{\text{a}}=1}^{60}\int_{0}^{2\pi }d\Phi \int_{-\infty }^{+\infty }dZ\;v(|{\vec{r}}_{R}-R^{-1}(\alpha ,\beta ,\gamma ){\vec{r}}_{\Lambda_{\text{a}}}|)$$

Expression (3) does not make use of the molecule’s symmetry; its numerical implementation requires the use of explicit Euler rotation matrices and is computationally heavy. To exploit the symmetries of both the molecule and the surrounding tube, we proceed as follows. First, we introduce spherical coordinates for the atoms of the molecule, *r⃗*_Λ_a__ = (*r*_Λ_a__ sin *θ*_Λ_a__ cos *φ*_Λ_a__*, r*_Λ_a__ sin *θ*_Λ_a__ sin *φ*_Λ_a__*, r*_Λ_a__ cos *θ*_Λ_a__), and rewrite the interaction energy for a molecule in the standard orientation as

(4) $$V=\sigma R\sum_{\Lambda_{\text{a}}=1}^{60}w(R;r_{\Lambda_{\text{a}}},\theta_{\Lambda_{\text{a}}},\varphi_{\Lambda_{\text{a}}})$$

with

(5) $$w(R;r_{\Lambda_{\text{a}}},\theta_{\Lambda_{\text{a}}},\varphi_{\Lambda_{\text{a}}})=\int_{0}^{2\pi }d\Phi \int_{-\infty }^{\infty }dZ\;v(|{\vec{r}}_{R}-{\vec{r}}_{\Lambda_{\text{a}}}|)$$

The distance *|r⃗_R_* − *r⃗*_Λ_a__*|* reads

(6) $$|{\vec{r}}_{R}-{\vec{r}}_{\Lambda_{\text{a}}}|=\sqrt{R^{2}+Z^{2}+r_{\Lambda_{\text{a}}}^{2}-2Rr_{\Lambda_{\text{a}}}\text{cos}(\Phi -\varphi_{\Lambda_{\text{a}}})\text{sin}\theta_{\Lambda_{\text{a}}}-2Zr_{\Lambda_{\text{a}}}\text{cos}\theta_{\Lambda_{\text{a}}}}$$

Only the difference of Φ and *φ*_Λ_a__ enters expression (6), as the argument of the function cos. Therefore, the quantity *w* is independent of *φ*_Λ_a__, since a change of variables Φ′ = Φ − *φ*_Λ_a__ eliminates *φ*_Λ_a__ from the expression for *|r⃗_R_* − *r⃗*_Λ_a__*|* and since ∫*_φ_*_Λ_a__^2^*^π^*^+^*^φ^*^Λ_a_^ *d*Φ′*f*(Φ′) = ∫_0_^2^*^π^* *d*Φ′*f*(Φ′) for a function *f* with periodicity 2*π*. For a C_60_ molecule, all C atoms have the same radial coordinate *r*_Λ_a__ ≡ *r*_a_, which can therefore be considered a constant rather than a variable in expressions (4) – (6). Hence, we end up with the equations

(7) $$V=\sigma R\sum_{\Lambda_{\text{a}}=1}^{60}w(R;\theta_{\Lambda_{\text{a}}})$$

(8) $$w(R;\theta_{\Lambda_{\text{a}}})=\int_{0}^{2\pi }d\Phi^{\prime }\int_{-\infty }^{\infty }dZ\;v(d(R;Z,\Phi^{\prime },\theta_{\Lambda_{\text{a}}}))$$

(9) $$d(R;Z,\Phi^{\prime },\theta_{\Lambda_{\text{a}}})=\sqrt{R^{2}+Z^{2}+r^{2}-2Rr\text{cos}\Phi^{\prime }\text{sin}\theta_{\Lambda_{\text{a}}}-2Zr\text{cos}\theta_{\Lambda_{\text{a}}}}$$

The quantity *w*(*R; θ*_Λ_a__), taken as a function of *θ*_Λ_a__, can be expanded into *m* = 0 spherical harmonics:

(10) $$w(R;\theta_{\Lambda_{\text{a}}})=\sum_{l=0}^{\infty }v_{l}(R)Y_{l}^{0}(\theta_{\Lambda_{\text{a}}})$$

(11) $$\begin{array}{l} v_{l}(R)=2\pi \int_{0}^{\pi }\text{sin}\theta d\theta \;Y_{l}^{0}(\theta )w(R;\theta )\\ =2\pi \int_{0}^{\pi }\text{sin}\theta d\theta \int_{0}^{2\pi }d\Phi^{\prime }\int_{-\infty }^{\infty }dZ\;Y_{l}^{0}(\theta )v(d(R;Z,\Phi^{\prime },\theta )) \end{array}$$

Here, we use the Bradley and Cracknell spherical harmonics (Ref. [11]). Upon rotation, spherical harmonics transform into linear combinations of spherical harmonics. One has

(12) $$R(\alpha ,\beta ,\gamma )Y_{l}^{m}(\theta ,\varphi )=\sum_{n=-l}^{l}D_{n,m}^{l}(\alpha ,\beta ,\gamma )Y_{l}^{n}(\theta ,\varphi )$$

The rotation operator ℜ(*α, β, γ*) has been introduced before; the quantities 
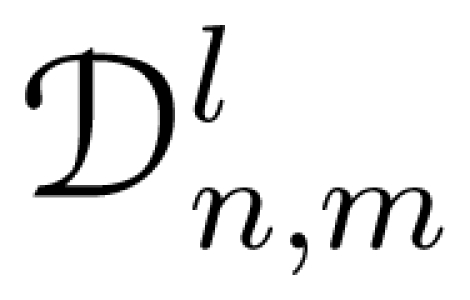
 (*α, β, γ*) are the Wigner 
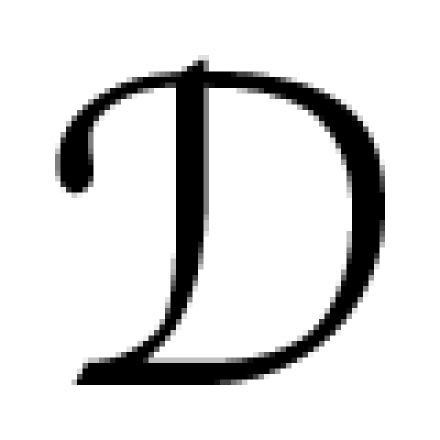
-functions. In the present case, cylindrical symmetry implies *m* = 0, for which the *α*-independent Wigner 
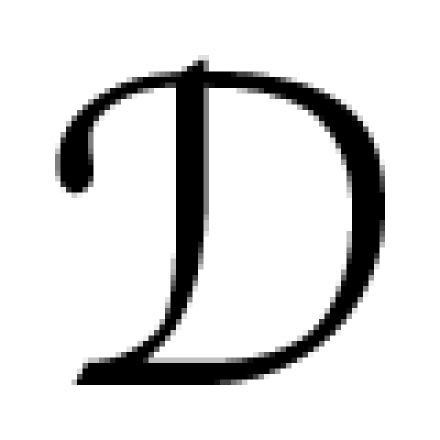
-functions reduce to

(13) $$D_{n,m=0}^{l}(\beta ,\gamma )=\sqrt{\frac{4\pi }{2l+1}}{\left[Y_{l}^{n}(\beta ,\gamma )\right]}^{*}$$

Collecting the previous equations results in the following potential energy expression for a rotated molecule:

(14) $$\begin{array}{l} V(\alpha ,\beta ,\gamma )=\sigma R\sum_{\Lambda_{\text{a}}=1}^{60}\sum_{l=0}^{\infty }v_{l}(R)R(\alpha ,\beta ,\gamma )Y_{l}^{0}(\theta_{\Lambda_{\text{a}}})\\ =\sigma R\sum_{l=0}^{\infty }v_{l}(R)\sum_{n=-l}^{l}D_{n,m=0}^{l}(\beta ,\gamma )\sum_{\Lambda_{\text{a}}=1}^{60}Y_{l}^{n}(\theta_{\Lambda_{\text{a}}},\varphi_{\Lambda_{\text{a}}})\equiv V(\beta ,\gamma ) \end{array}$$

The potential energy *V* (*β, γ*) depends on the molecule’s orientation and on the tube’s radius; it can therefore be considered as a potential energy field (nanotube field) set up by the surrounding tube and experienced by the molecule. Note that there is no *α*-dependence since the initial rotation ℜ*_z_*(*α*) about the *z*-axis has no effect on the interaction energy—a consequence of the “smooth-tube approximation”.

So far, only the cylindrical symmetry—the site symmetry—has been used. The molecular symmetry is accounted for by the distribution of C atoms. We introduce atomic form factors *c_l_^n^*,

(15) $$c_{l}^{n}=\sum_{\Lambda_{a}=1}^{60}Y_{l}^{n}(\theta_{\Lambda_{a}},\varphi_{\Lambda_{a}})$$

molecular shape factors *g_l_*,

(16) $$g_{l}=\sqrt{\sum_{n=-l}^{l}{\left(c_{l}^{n}\right)}^{2}}$$

and normalised atomic form factors *α_l_^n^*,

(17) $$\alpha_{l}^{n}=\frac{c_{l}^{n}}{g_{l}}$$

Icosahedral molecular symmetry implies that *c_l_^n^* differs from zero only for *n* even and *l* = 0*,* 6*,* 10*,* 12*, . . .*. The non-vanishing *g_l_* and *α_l_^n^* values are tabulated in Table 1 up to *l* = 12. We can now rewrite *V* (*β, γ*) as

(18) $$V(\beta ,\gamma )=\sigma R\sum_{l=0,6,10,12,\ldots }v_{l}(R)g_{l}U_{l}(\beta ,\gamma )$$

where

(19) $$U_{l}(\beta ,\gamma )=\sum_{n=-l}^{l}\alpha_{l}^{n}D_{n,0}^{l}(\beta ,\gamma )$$

are molecular-and-site-symmetry-adapted rotator functions (SARFs). Rotator functions, originally introduced by James and Keenan [2], are the appropriate variables for the description of orientationaldependent properties of molecules in crystals [3,4]. They account for the symmetry of the molecule and the symmetry of the crystal site point group. In the present case the crystal site symmetry is the *D_∞h_* symmetry of the (smooth) nanotube. The cylindrical site symmetry has the consequence that the Wigner 
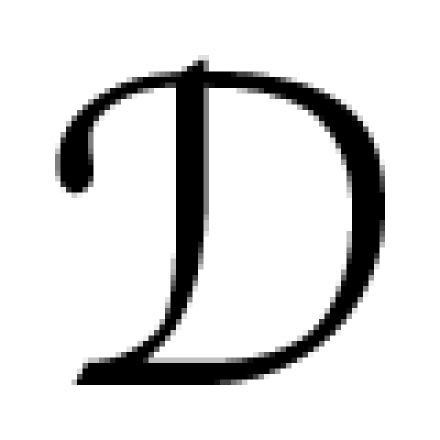
-functions are linear combinations of spherical harmonics [Equation (13)], and that the rotator functions 
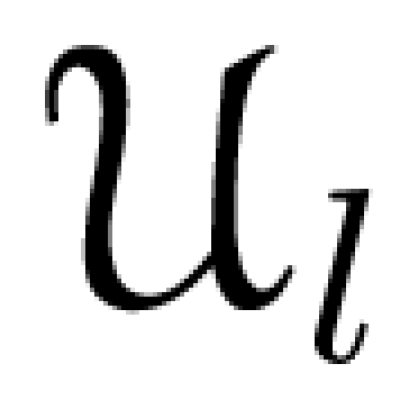
(*β, γ*) can be easily implemented:

(20) $$U_{l}(\beta ,\gamma )=\sqrt{\frac{4\pi }{2l+1}}\sum_{n=-l}^{l}\alpha_{l}^{n}{\left[Y_{l}^{n}(\beta ,\gamma )\right]}^{*}$$

The symmetry of a C_60_ molecule implies some restrictions on the atomic form factors. The combination of a center of inversion and the (*x, z*)- and (*x, y*)-planes being mirror planes results in the follow properties:

(21a) $$c_{l}^{n}=0\;\text{if }l\;\text{odd}$$

(21b) $${\left(c_{l}^{n}\right)}^{*}=c_{l}^{n}$$

(21c) $$c_{l}^{-n}=c_{l}^{n}$$

(21d) $$c_{l}^{n}=0\;\text{if }l\;\text{even and }n\;\text{odd}$$

The same relations hold for the normalized molecular form factors *α_l_^n^*. In particular, it follows that the rotator functions are real functions since *α_l_*^−^*^n^* = *α_l_^n^* and [*Y_l_^n^* (*β, γ*)]^*^ = *Y_l_*^−^*^n^* (*β, γ*).

In summary, as a numerically much more efficient alternative to Equation (3) involving explicit coordinate transformations (Euler rotations), one can maximally exploit the symmetry of both the molecule and its environment by first calculating the atomic form factors *c_l_^n^* [Equation (15)], molecular shape factors *g_l_* [Equation (16)], and normalised atomic form factors *α_l_^n^* [Equation (17)]. The latter then serve as coefficients in linear combinations of spherical harmonics defined as SARFs [Equation (20)]. These coefficients have only to be calculated once. For a given tube radius *R*, the expansion coefficients *v_l_*(*R*) are calculated by numerical integration [Equation (11)]. The evaluation of a few leading terms of [Equation (18)] then serves as an excellent approximation for the molecule’s potential energy *V* (*β, γ*).

For actual calculations, a potential function and potential parameters have to be specified. In Refs. [12] and [13], a Born–Mayer–van der Waals C-C pair potential,

(22) $$v(d)=C_{1}e^{-C_{2}d}-\frac{B}{d^{6}}$$

was introduced for studying C_60_-C_60_ interactions in C_60_-fullerite (solid buckminsterfullerene); it led to a crystal field potential and a structural phase transition temperature [14,15] in good agreement with experiments. Using the potential constants *C*_1_ = 3.24 *×* 10^7^ K *× k*_B_, *C*_2_ = 3.6 Å^−1^ and *B* = 4.579 *×* 10^5^ K *× k*_B_ · Å^6^ of Refs. [6] and [7] results in the *v_l_*(*R*) coefficients, obtained via numerical integration of expression (11), shown in Table 2. The amplitude of these coefficients decreases with increasing *l*. More indicative are the weighted coefficients *g_l_v_l_*(*R*), also given in Table 2. They reveal the relative importance of the contributing *l* terms. The *l* = 12 terms clearly contribute much less than the *l* = 6 terms— the lowest-order terms introducing (*β, γ*)-dependence, but the *l* = 10 contribution obviously plays an important role for the *R* = 6.0 and *R* = 7.0 cases.

**Figure 2:**
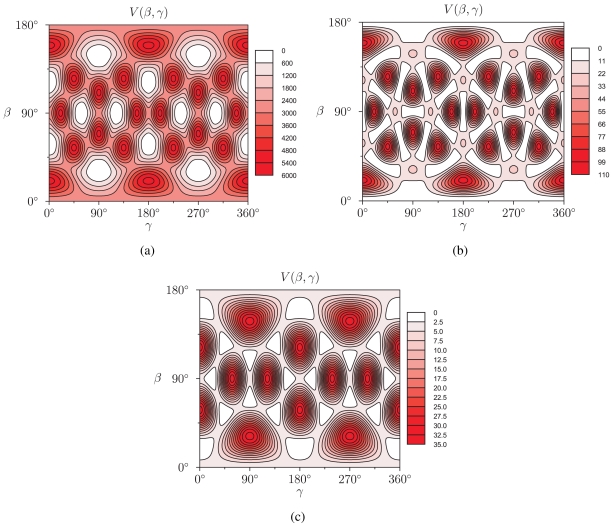
Nanotube field *V* (*β, γ*) of a C_60_ molecule in a CNT with radius (a) *R* = 6.0 Å, (b) *R* = 7.0 Å and (c) *R* = 8.0 Å, in units K *× k*_B_. The absolute minima have been subtracted so that the local energy minima lie at zero.

Having calculated the quantities *g_l_*, *v_l_*(*R*) and *α_l_^n^* allows to construct the rotator functions 
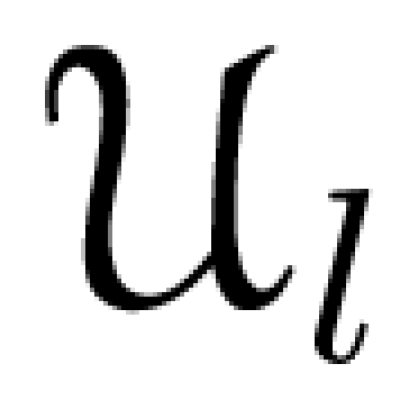
(*β, γ*) and to evaluate expression (18). In Figure 2, we show the results of *V* (*β, γ*) for *R* = 6.0 Å, *R* = 7.0 Å and *R* = 8.0 Å for a calculation up to *l* = 12. For *R* = 6.0 Å, there are 12 local minima, corresponding to the twelve equivalent molecular orientations where two opposing pentagons of the C_60_ molecule are perpendicular to the *z*-axis. The 20 maxima correspond to orientations where hexagons are perpendicular to the *z*-axis. (The “soccer-ball structure” indeed features 12 pentagons and 20 hexagons.) At *R* = 7.0 Å, there are 30 local minima: they correspond to the molecular configurations where opposing double bonds are perpendicular to the tube’s long axis (*z*-axis). The potential energy is maximal when a hexagon is perpendicular to the *z*-axis. Finally, at *R* = 8.0 Å, minima and maxima correspond to hexagons and pentagons perpendicular to the *z*-axis, respectively.

When comparing the nanotube fields shown in Figure 2 to the (*β, γ*)-maps obtained via the exact formula with explicit coordinate transforms [Equation (3)], there is no visual difference: the SARFs expansion up to *l* = 12 is an excellent approximation. Using the same integration routines, the direct calculations took a few hours each, however, while the evaluation of the *v_l_*(*R*) coefficients and the SARFs expansion was a matter of seconds. Therefore, in addition to providing physical and mathematical understanding of the symmetries present in the system, SARFs have a considerable computational advantage.

### 2.2. Discussion

The important feature in the construction of an expansion into SARFs is the vanishing of several atomic form factors *c_l_^n^* (*α_l_^n^*). This is a direct consequence of the symmetry of the considered molecule. In terms of group theory, only certain linear combinations of spherical harmonics *Y_l_^n^* (*β, γ*) belong to the unit representation (*A*_1_*_g_* irreducible representation) of the molecular symmetry group and contribute to the expansion of *V* (*β, γ*) into SARFs. For a cylindrical tube there is no *α*-dependence, and the expansion into SARFs is in fact nothing but an expansion into linear combinations of spherical harmonics *Y_l_^n^* (*β, γ*) belonging to the unit representation.

### 2.3. Extension to clusters with different types of interaction centers

Often, several types of molecular sites are treated as interaction centers (ICs). In the case of a molecule consisting of different types of atoms, every atomic type interacts differently with the surrounding nanotube, which can be accounted for by using different potential constants (or even different potential functions). The pair potential *v*(*d*) and the expansion coefficients *v_l_*(*R*) then become type-dependent:

(23) $$v_{l}^{\text{t}}(R)=2\pi \int_{0}^{\pi }\text{sin}\theta d\theta \int_{0}^{2\pi }d\Phi^{\prime }\int_{-\infty }^{\infty }dZ\;Y_{l}^{0}(\theta )v^{\text{t}}(d(R;Z,\Phi^{\prime },\theta ))$$

Here, the superscript ^t^ stands for the IC type. The ICs need not only be atoms; in the case of C_60_ molecules, it is customary to place ICs on bonds. For a C_60_ molecule, double bonds (fusing hexagons) and single bonds (fusing hexagons and pentagons) are considered, and labeled t = db and t = sb, respectively. (By bonds, the midpoints of bonds are understood.) These additional ICs were originally introduced to account for variations in the charge distribution of a C_60_ molecule [13,16]. For each of the IC types, t = a, db, sb, the atomic form factors

(24) $$c_{l}^{\text{t},n}=\sum_{\Lambda_{\text{t}}=1}^{60}Y_{l}^{n}(\theta_{\Lambda_{\text{t}}},\varphi_{\Lambda_{\text{t}}})$$

the molecular shape factors

(25) $$g_{l}^{\text{t}}=\sqrt{\sum_{n=-l}^{l}{\left(c_{l}^{\text{t},n}\right)}^{2}}$$

and the normalised atomic form factors

(26) $$\alpha_{l}^{\text{t},n}=\frac{c_{l}^{\text{t},n}}{g_{l}^{\text{t}}}$$

have to be calculated. Here, *θ*_Λ_t__ and *φ*_Λ_t__ stand for the polar and azimuthal angles of the ICs of type t, labeled Λ_t_ = 1*, . . . ,N*_t_, with *N*_t_ the number of ICs of type t (*N*_a_ = 60, *N*_db_ = 30 and *N*_sb_ = 60). Remarkably, it turns out that

(27) $$\alpha_{l}^{\text{t},n}=\xi_{l}^{\text{t}}\alpha_{l}^{\text{a},n}$$

with *ξ_l_*^t^ = *±*1. It is therefore useful to introduce modified molecular shape factors

(28) $${\tilde{g}}_{l}^{\text{t}}=\xi_{l}^{\text{t}}g_{l}^{\text{t}}$$

so that

(29) $$c_{l}^{\text{t},n}={\tilde{g}}_{l}^{\text{t}}\alpha_{l}^{n}$$

where *α_l_^n^* ≡ *α_l_*^a^*^,n^*. This allows to write the following generalised expression for *V* (*β, γ*):

(30) $$V(\beta ,\gamma )=\sigma R\sum_{l=0,6,10,12,\ldots }\sum_{\text{t}=\text{a},\text{db},\text{sb}}v_{l}^{\text{t}}(R){\tilde{g}}_{l}^{\text{t}}U_{l}(\beta ,\gamma )$$

Note that the same SARFs as before [Equation (19)] can be used.

The nanotube fields of a C_60_ molecule arising from the extended interaction model described in the present subsection do not differ qualitatively from the ones shown in Figure 2. For full details, we refer to Ref. [8].

The manifestation of potential energy landscapes as in Figure 2 as a consequence of molecular symmetry and cylindrical confinement and their dependence on the tube radius *R* has important implications on the physical properties of a “peapod”—a chain of several molecules encapsulated in a CNT. In particular, the peapod system of C_60_ molecules encapsulated in a CNT displays unusual dynamical behavior as demonstrated by different experimental techniques: inelastic neutron scattering [17], nuclear magnetic resonance [18,19] and high-pressure near-infrared Raman scattering [20]. Obviously, for a realistic description of peapods, molecule-molecule interactions have to be taken into account as well. For typical tube radii (*R ~* 7 Å) of C_60_ peapod samples, though, the intermolecular interactions are several orders of magnitude smaller than the molecule-tube interaction [8] and do therefore not significantly influence the molecules’ optimal orientation with respect to the surrounding CNT. A discussion of the dynamics of the molecules in a C_60_-peapod is beyond the scope of the present paper, however — we recall that our purpose is to provide the mathematical framework for the effective exploitation of the molecular and the environmental symmetry for calculating potential energies. For details, we refer to the relevant experimental [17,18,19,20] and theoretical [6,7,8] literature.

### 2.4. General formulation for non-spherical clusters of interaction centers

In the foregoing we have introduced SARFs for a C_60_ molecule, displaying icosahedral symmetry, with atoms, double and single bonds considered as three different types of ICs. A special feature of the C_60_ molecule is that for each IC type, the radial coordinates of the ICs are equal (dependent on t, not on Λ_t_): *r*_Λ_a__ ≡ *r*_a_, *r*_Λ_db__ ≡ *r*_db_, *r*_Λ _sb__ ≡ *r*_sb_. This does not hold for all symmetries, however. The general formulation of a molecule’s nanotube field *V* (*β, γ*) has to take this into account.

**Figure 3:**
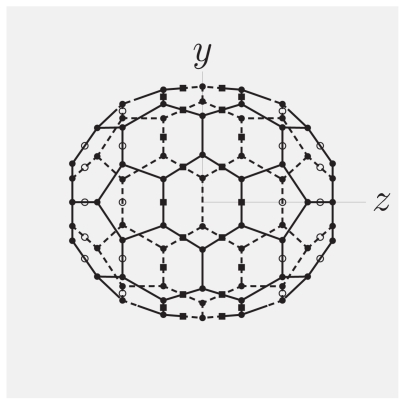
Projection of a C_70_ molecule in the standard orientation on the (*y, z*)-plane. Apart from atoms (dots), midpoints of certain bonds in the “cap” and “belt” regions are considered as ICs as well— D-centers (circles) and I-centers (squares), respectively.

To fix ideas, we take the example of a C_70_ molecule, which has an ellipsoidal shape and*D*_5_*_h_* symmetry. (All formulas of this subsection will hold in general, though.) A popular IC cluster model for C_70_ features the 70 carbon atoms (t = a), 20 so-called D-centers on bonds near the top and bottom of the molecule (t = D) and 30 so-called I-centers in the “equatorial zone” of the molecule as ICs (Figure 3). The essential step to develop appropriate SARFs is to group ICs with the same value of the radial coordinate *r*_Λ_t__. In the case of a C_70_ molecule, ICs with the same *|z*_Λ_t__*|* value have the same *r*_Λ_t__ value. Therefore, we can think of layers of ICs having the absolute value of their *z*-coordinate in common. We use the term ‘layers’ in an abstract way and let it refer to a group of ICs with equal *r*_Λ_t__ values. We label the layers by an index *λ*_t_, and the ICs within layer *λ*_t_ by an index *ν_λ_*__t__. This results in a compound index

(31) $$\Lambda_{\text{t}}\equiv (\lambda_{\text{t}},\nu_{\lambda_{\text{t}}})$$

to address IC Λ_t_. Introducing the layer-dependent analogues of Equations (15) – (17),

(32a) $$c_{l}^{\text{t},n}(\lambda_{\text{t}})=\sum_{\nu_{\Lambda_{\text{t}}}}Y_{l}^{n}(\theta_{\Lambda_{\text{t}}},\varphi_{\Lambda_{\text{t}}})$$

(32b) $$g_{l}^{\text{t}}(\lambda_{\text{t}})=\sqrt{\sum_{n=-l}^{l}{\left(c_{l}^{\text{t},n}(\lambda_{\text{t}})\right)}^{2}}$$

(32c) $$\alpha_{l}^{\text{t},n}(\lambda_{\text{t}})=\frac{c_{l}^{\text{t},n}(\lambda_{\text{t}})}{g_{l}^{\text{t}}(\lambda_{\text{t}})}$$

and the layer-dependent SARFs

(33) $$U_{l}^{\text{t}}(\lambda_{\text{t}};\beta ,\gamma )=\sum_{n=-l}^{l}\alpha_{l}^{\text{t},n}(\lambda_{\text{t}})D_{n,0}^{l}(\beta ,\gamma )$$

results in the following expression for the molecule’s nanotube field:

(34) $$V(\beta ,\gamma )=\sigma R\sum_{l=0}^{\infty }v_{l}^{\text{t}}(R)g_{l}^{\text{t}}U_{l}^{\text{t}}(\lambda_{\text{t}};\beta ,\gamma )$$

Equation (34) is the most general form of the SARFs expansion for a molecule placed on the long axis of a CNT. It takes into account different IC types and the non-spherical distribution of ICs. Note that there is no proportionality rule like Equation (29) in the case of “layered” structures (different *r*_Λ_t__ values for IC type t). Results for the C_70_ molecule’s nanotube fields and their physical implications can be found in Ref. [9].

### 2.5. Example #2

**Figure 4:**
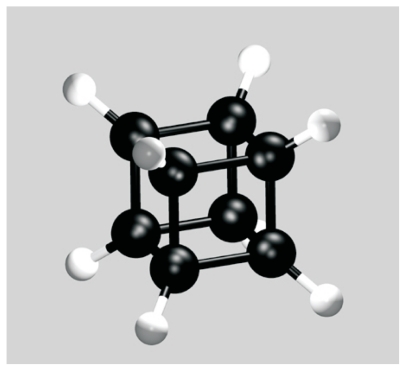
A C_8_H_8_ molecule.

We now apply the SARFs procedure to an example with cubic molecular symmetry. Cubane, C_8_H_8_, has eight carbon atoms arranged on the corners of a cube to each of which a hydrogen atom is bound (Figure 4). While its chemical synthesis dates back to 1964 [21], it has gained renewed interest after the successful synthesis of fullerene-cubane, C_60_.C_8_H_8_, a remarkable molecular crystal consisting of icosahedral (*I_h_*) and cubic (*O_h_*) molecules with stoichiometry 1:1 [22].

We consider a cubane molecule encapsulated in a CNT with radius *R*; it is intended as a generic example of a cubic molecule inserted into a nanotube. An example of an actual molecule with *O_h_* symmetry that has successfully been encapsulated in a nanotube is octasilesquioxane, Si_8_H_8_O_12_ [23,24].

We model the cubane molecule as a simple cubic cluster of 8 ICs placed on the H atoms and define the standard orientation (*α* = *β* = *γ* = 0) as the orientation where the cube’s faces are parallel to the coordinate planes. The ICs then have coordinates (*±a,±a,±a*), (*±a,±a, a*), (*±a, a,±a*) and (*a,±a,±a*) with *a* = 1.4139 Å. First, the atomic form factors *c_l_^n^* and the derived quantities *g_l_* and *α_l_^n^* have to be determined [Equations (15) – (17)]. In Table 3 we show the non-zero *g_l_* and *α_l_^n^* coefficients up to *l* = 12. The symmetry relations (21a) – (21d) are also valid for cubic symmetry. In addition, *c_l_^n^* (*α_l_^n^*) coefficients vanish if *n* is not a multiple of 4:

(35) $$c_{l}^{n}=0\;\text{if }n\notin 4Z$$

The lowest non-zero *l*-value yielding non-vanishing *c_l_^n^* coefficients is *l* = 4. This is a well-known result from group theory; the *l* = 4 rotator function

(36) $$U_{4}(\beta ,\gamma )=\sqrt{\frac{4\pi }{9}}[\alpha_{4}^{4}Y_{4}^{4}(\beta ,\gamma )+\alpha_{4}^{0}Y_{4}^{0}(\beta ,\gamma )+\alpha_{4}^{4}Y_{4}^{-4}(\beta ,\gamma )]$$

is proportional to the cubic harmonic *K*_4_(*β, γ*). The next non-zero terms in the SARFs expansion have *l* = 6*,* 8*,* 10*,* 12*, . . .*.

**Figure 5:**
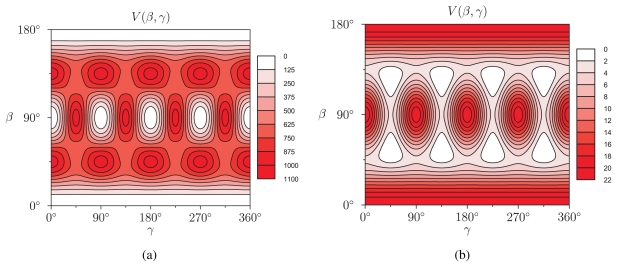
Nanotube field *V* (*β, γ*) of a C_8_H_8_ molecule in a CNT with radius (a) *R* = 5.0 Å and (b) *R* = 8.0 Å, in units K *× k*_B_. The absolute minima have been subtracted so that the local energy minima lie at zero.

For the pair interaction potential *v*(*d*) we take the Lennard-Jones potential used for modeling cubanefullerene interactions in C_60_.C_8_H_8_ [25]:

(37) $$v(d)=4ɛ\left[{\left(\frac{\sigma }{d}\right)}^{12}-{\left(\frac{\sigma }{d}\right)}^{6}\right]$$

with *ɛ* = 16.733 K *× k*_B_ and *σ* = 2.895 Å. For two radii, *R* = 5.0 Å and *R* = 7.0 Å, we first calculate the expansion coefficients *v_l_*(*R*) [Equation (11)]. They are shown in Table 4 up to *l* = 12, together with the weighted expansion coefficients *g_l_v_l_*(*R*). The magnitude of *g_l_v_l_*(*R*) decreases rapidly with increasing *l*. The *R* = 5.0 Å and *R* = 7.0 Å nanotube fields, calculated via the SARFs expansion up to *l* = 12, Equation (18), are shown in Figure (5). Both display cubic symmetry, but the local minima and maxima are located differently. For *R* = 5.0 Å, 6 equivalent minima can be distinguished. They correspond to the 6 realisations of the standard orientation where the cubane molecule’s faces are aligned with the coordinate planes. There are 12 maxima, they correspond to the orientations where the *z*-axis (long axis of the tube) intersects the midpoints of two opposing edges of the cube. At *R* = 7.0 Å, the local minima have become local maxima. There are 8 local minima, corresponding to orientations where the tube’s long axis intersects two opposing vertices of the cube. These findings are relevant for explaining actual experimental results; Si_8_H_8_O_12_ molecules (of cubic symmetry) inserted in CNTs with radii *R ≈* 6 Å - 7 Å self-assemble into Si_4_*_n_*H_8_O_8_*_n_*_−4_ ladder-like structures [26]. We argue that the optimal orientations found here are a necessary prerequisite for the formation of the experimentally observed chemical bonds between neighboring octasilesquioxane monomers.

## 3. Conclusions

We have outlined the construction of SARFs for molecules of any symmetry in cylindrical confinement. The molecules are taken as discrete clusters of ICs, labeled Λ_t_, of different types, labeled t. In general, SARFs 
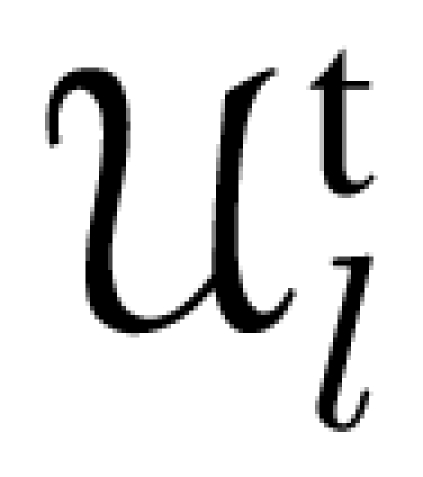
 (*λ*_t_; *β, γ*) are obtained via Equation (33), where the index *λ*_t_ groups ICs having the same radial coordinate *r*_Λ_t__ ≡ *r_λ_*__t__ (layers of ICs). The SARFs are type- and layer-dependent. In some special cases, e.g., for spherical clusters like C_60_, type-independent SARFs can be constructed. The main consequence of the cylindrical site symmetry is the SARFs’ independence on the Euler angle *α*. The molecule-tube interaction energy (nanotube field) is conveniently obtained as an expansion into SARFs, Equation (34), where the expansion coefficients *v_l_*^t^ (*R*) are obtained via numerical integrations [Equation (23)] involving the pair potentials *v*_t_(*d*). It turns out that a limited number of terms (typically up to *l* = 12) provides an excellent approximation to the exact expression [Equation (3)] with explicit coordinate transforms. For concrete examples, the SARFs expansion has proven to be computationally much more efficient: the calculations of a C_60_ molecule’s nanotube field take less than 1000 times the time for the direct calculation.

Knowledge of the nanotube field of a molecule encapsulated in a CNT immediately allows to identify stable molecular orientations. In the case of C_60_ molecules, depending on the tube radius *R*, different configurations are energetically favorable: pentagons (*R* = 6.0 Å), double bonds (*R* = 7.0 Å) or hexagons (*R* = 8.0 Å) perpendicular to the tube’s long axis. For cubic molecules, an example of which is C_8_H_8_, we also find different regimes. For small radii, the cube’s faces are aligned to the crystal planes, while for large radii, two opposing edges are intersected halfway by the tube’s long axis of the tube.

The computational efficiency for nanotube field calculations is one of the main advantages of using SARFs. There are, however, many more situations in which SARFs are useful, especially in the context of orientational order-disorder phase transitions in molecular crystals (see e.g. Ref. [27] for a treatment of the *Fm*3̄*m* → *Pa*3̄ phase transition in solid C_60_). The general theoretical framework of SARFs as described by Michel and Parlinski [4] is readily applicable to the one-dimensional crystals resulting from inserting molecules in CNTs. For example, the thermal averages

(38) $$\langle U_{l}^{\text{t}}(\lambda_{\text{t}})\rangle =\frac{\int_{0}^{\pi }\text{sin}\beta d\beta \int_{0}^{2\pi }d\gamma \;U_{l}^{\text{t}}(\lambda_{\text{t}};\beta ,\gamma )e^{-\frac{V(\beta ,\gamma )}{k_{\text{B}}T}}}{\int_{0}^{\pi }\text{sin}\beta d\beta \int_{0}^{2\pi }d\gamma \;e^{-\frac{V(\beta ,\gamma )}{k_{\text{B}}T}}}$$

can play the role of order parameters of second-order orientational phase transitions and are also are quantities relevant for the interpretation of Raman and/or infra-red spectroscopic measurements.

Throughout the paper, we have worked under the smooth-tube approximation, neglecting the actual honeycomb network of carbon atoms of the CNT. As has been shown by comparing the results of both the smooth-tube approach and calculations taking the discrete structure of a CNT into account, this is a valid approximation [8,9]. Another assumption has been that the molecule is located on the tube’s long axis. While this is plausible for small tube radii because of the strong repulsion between the molecule and the surrounding tube wall, one expects a shift Δ*r* away from the tube’s axis from a certain radius onwards. This is indeed the case; for C_60_ and higher (tubular) fullerene molecules, the energetically favorable position is off-axis from *R ≈* 7 Å [6,7,28] onwards. For small deviations from the on-axis position, a Taylor expansion into powers of the off-axis shift Δ*r* in combination with an expansion into SARFs can provide a generalization of the on-axis treatment given here. However, this is beyond the scope of the present paper.

## Figures and Tables

**Table 1:** Atomic form factors $c_{l}^{n}$, molecular shape factors *g_l_*, and normalised atomic form factors $\alpha_{l}^{n}$ for *I_h_* symmetry.

*l*	*g_l_*	*n*	$\alpha_{l}^{n}$
0	16.9257	0	1

6	2.6365	0	−0.2073
6		2	−0.4750
6		4	0.3878
6		6	0.3202

10	19.2982	0	0.3545
10		2	−0.2880
10		4	−0.3572
10		6	−0.0565
10		8	−0.4251
10		10	0.2069

12	9.0051	0	−0.4145
12		2	−0.1179
12		4	−0.1830
12		6	0.4635
12		8	−0.0738
12		10	−0.2924
12		12	−0.2469

**Table 2:** Expansion coefficients *v_l_* *(R*) and weighted expansion coefficients *g_l_v_l_* *(R*) for *R* = 6.0 Å, *R* = 7.0 Å and *R* = 8.0 Å, obtained with Born–Mayer–van der Waals potential (22), in units K *× k*_B_ · Å.

*R*	*v*_0_(*R*)	*v*_6_(*R*)	*v*_10_(*R*)	*v*_12_(*R*)
6.0 Å	−2201.02	−833.92	−53.79	7.87
7.0 Å	−2151.95	−7.81	−1.99	0.36
8.0 Å	−886.63	4.23	−0.04	0.01

*R*	*g*_0_*v*_0_(*R*)	*g*_6_*v*_6_(*R*)	*g*_10_*v*_10_(*R*)	*g*_12_*v*_12_(*R*)

6.0 Å	−37253.82	−2198.65	−1038.13	62.97
7.0 Å	−36423.20	−20.58	−38.49	2.85
8.0 Å	−15006.86	11.14	−0.71	0.09

**Table 3:** Atomic form factors $c_{l}^{n}$, molecular shape factors *g_l_,* and normalised atomic form factors $\alpha_{l}^{n}$ for *O_h_* symmetry.

*l*	*g_l_*	*n*	$\alpha_{l}^{n}$
0	2.2568	0	1

4	3.4473	0	−0.7638
4		4	−0.4564

6	5.1143	0	0.3536
6		4	−0.6614

8	1.9797	0	0.7181
8		4	0.2700
8		8	0.4114

10	6.7237	0	−0.4114
10		4	0.4146
10		8	0.4934

12	4.6866	0	0.0919
12		4	−0.3625
12		8	0.5977
12		12	−0.0849

**Table 4:** Expansion coefficients *v_l_* *(R*) and weighted expansion coefficients *g_l_v_l_* *(R*) for *R* = 5.0 Å and *R* = 7.0 Å, obtained with Lennard-Jones potential (37), in units K *× k*_B_ · Å.

*R*	*v*_0_(*R*)	*v*_4_(*R*)	*v*_6_(*R*)	*v*_8_(*R*)	*v*_10_(*R*)	*v*_12_(*R*)
5.0 Å	−474.28	106.87	−49.25	14.53	−3.38	0.67
7.0 Å	−103.26	−1.89	0.11	0.00	0.00	0.00

*R*	*g*_0_*v*_0_(*R*)	*g*_4_*v*_4_(*R*)	*g*_6_*v*_6_(*R*)	*g*_8_*v*_8_(*R*)	*g*_10_*v*_10_(*R*)	*g*_12_*v*_12_(*R*)

5.0 Å	−1070.37	368.41	−251.86	28.76	−22.71	3.14
7.0 Å	−233.04	−6.53	0.55	−0.01	0.00	0.00
